# Alternative scoring methods of fusarium head blight resistance for genomic assisted breeding

**DOI:** 10.3389/fpls.2022.1057914

**Published:** 2023-01-11

**Authors:** J. Garcia-Abadillo, L. Morales, H. Buerstmayr, S. Michel, M. Lillemo, J. Holzapfel, L. Hartl, D. Akdemir, H. F. Carvalho, J. Isidro-Sánchez

**Affiliations:** ^1^ Department of Biotechnology and Plant Biology - Centre for Biotechnology and Plant Genomics (CBGP) - Universidad Politécnica de Madrid (UPM), Madrid, Spain; ^2^ Department of Agrobiotechnology, Institute of Biotechnology in Plant Production, University of Natural Resources and Life Sciences Vienna (BOKU), Tulln an der Donau, Austria; ^3^ Department of Plant Sciences, Norwegian University of Life Sciences (NMBU), Ås, Norway; ^4^ Secobra Saatzucht GmbH, Moosburg, Germany; ^5^ Bavarian State Research Center for Agriculture, Institute for Crop Science and Plant Breeding, Freising, Germany; ^6^ CIBMTR (Center for International Blood and Marrow Transplant Research), National Marrow Donor Program/Be The Match, Minneapolis, MN, United States

**Keywords:** genomic selection (GS), fusarium head blight (FHB), wheat, quantitative resistance, plant breeding, simulation and empirical evidence

## Abstract

Fusarium head blight (FHB) is a fungal disease of wheat (*Triticum aestivum*.L) that causes yield losses and produces mycotoxins which could easily exceed the limits of the EU regulations. Resistance to FHB has a complex genetic architecture and accurate evaluation in breeding programs is key to selecting resistant varieties. The Area Under the Disease Progress Curve (AUDPC) is one of the commonly metric used as a standard methodology to score FHB. Although efficient, AUDPC requires significant costs in phenotyping to cover the entire disease development pattern. Here, we show that there are more efficient alternatives to AUDPC (angle, growing degree days to reach 50% FHB severity, and FHB maximum variance) that reduce the number of field assessments required and allow for fair comparisons between unbalanced evaluations across trials. Furthermore, we found that the evaluation method that captures the maximum variance in FHB severity across plots is the most optimal approach for scoring FHB. In addition, results obtained on experimental data were validated on a simulated experiment where the disease progress curve was modeled as a sigmoid curve with known parameters and assessment protocols were fully controlled. Results show that alternative metrics tested in this study captured key components of quantitative plant resistance. Moreover, the new metrics could be a starting point for more accurate methods for measuring FHB in the field. For example, the optimal interval for FHB evaluation could be predicted using prior knowledge from historical weather data and FHB scores from previous trials. Finally, the evaluation methods presented in this study can reduce the FHB phenotyping burden in plant breeding with minimal losses on signal detection, resulting in a response variable available to use in data-driven analysis such as genome-wide association studies or genomic selection.

## 1 Introduction

Fusarium head blight (FHB), sometimes known as scab, is a global problem causing a great economic burden on the cereal industry due to its significant reductions in grain yield and quality Bottalico and Perrone (2002); Nganje et al. (2004); McMullen et al. (2012); Savary et al. (2019). Many Fusarium species are considered phytopathogenic fungi, but in bread and durum wheat the most serious FHB-causing agent is *Fusarium graminearum* Schwabe [telomorph: Gibberella zeae Schw. (Petch)]. Upon infection, several Fusarium species produce aggressive secondary metabolites, which lead to crop contamination such as, deoxynivalenol, nivalenol, T2 toxin, fumonisin, and mycoestrogen zearalenone, which have negative effects on human diet and on animal growth and fertility Placinta et al. (1999); Pestka and Smolinski (2005); Morimura et al. (2020).

Host resistance to FHB is quantitatively inherited with a complex genetic architecture and is usually split into components of resistance with partial overlapping control Schroeder (1963), that can be classified into several categories Mesterhazy (1995); Mesterházy et al. (1999). Type I resistance, refers to the initial resistance to the infection and it is usually measured as the percentage of infected heads in a plot as FHB incidence. Type II resistance, or FHB Severity, is the resistance to fungal spread and it can be measured as the percentage of infected spikelets in a head. Because of the global concern about poor wheat grain quality associated with harmful mycotoxins, resistance sources with type III for low DON accumulation and type IV for low Fusarium-damaged kernel (FDK) have recently attracted more wheat breeders’ attention. Fungal development depends on environmental factors such as moisture or temperature. Consequently, plants develop passive resistance mechanisms related to morphological and developmental traits Buerstmayr et al. (2020). FHB disease (combination of incidence and severity) is known to be partially correlated with plant height and flowering/anthesis date Jenkinson and Parry (1994); Buerstmayr et al. (2008).

Due to its complex genetic control and the fact that phenotyping is a labor and time-intensive process Van Sanford et al. (2001); Miedaner et al. (2012), alternatives to phenotypic selection such as marker-assisted selection (MAS) and genomic selection (GS) have been proposed as tools to select resistant varieties.

Genomic selection Meuwissen et al. (2001) has been promoted as an alternative to MAS in FHB resistance breeding Lorenz et al. (2012); Rutkoski et al. (2012); Arruda et al. (2015); Buerstmayr and Lemmens (2015); Mirdita et al. (2015); Hoffstetter et al. (2016); Herter et al. (2019); Buerstmayr et al. (2020); Larkin et al. (2020); Steiner et al. (2017; 2019). Instead of looking for statistically significant associations between markers and desired traits, GS uses genome-wide molecular marker information to predict the genetic value of lines that are genotyped but not phenotyped. This approach assumes that at least one marker is in linkage disequilibrium with each QTL underlying the trait of interest, regardless of the effect size Heffner et al. (2009). Many different statistical modelling approaches can be implemented on predicting FHB traits Heslot et al. (2012), nevertheless empirical results demonstrated that models often yield similar accuracy values when predicting FHB-related traits Arruda et al. (2016); Hoffstetter et al. (2016); Buerstmayr et al. (2020); Zhang et al. (2021).

The success of data-driven models such as MAS or GS relies on a good response variable or phenotypic value to properly capture genetic effects and/or complex interactions between genotypes and environments. Research for FHB resistance is often done through cooperative trial networks such as the annual CIMMYT FHB screening nurseries, the US wheat, and barley scab initiative, or the WheatSustain project (https://www.suscrop.eu/projects-first-call/wheatsustain), where data from multiple partners, locations and years are collected into a common dataset. These datasets are essential to developing robust and powerful models that yield reliable results and allow complex modelling such as genotype-by-environment interaction (GxE) Buerstmayr et al. (2020). However, the harmonization of the data and standardization of protocols is often a difficult task Akdemir et al. (2020). For example, plot designs, epidemic establishment origin (natural or artificial), or assessment protocols (number and timing of FHB evaluations) are key factors in field data acquisition. In this sense, scoring and comparing FHB resistance across trials should be done carefully.

The Area Under the Disease Progress Curve (AUDPC) is commonly used as a quantitative resistance phenotyping strategy because it integrates aspects related to host development and growth Jeger and Viljanen-Rollinson (2001). AUDPC is a particular application of Riemans’ssum where a discrete set of assessments are combined to approximate the definite integral of the function that would be ideally obtained if a set of infinite assessments (with an infinitesimal period between them) were available Thompson and Silverman (2008). From the growth point of view, biological processes are usually modelled using S-shape, sigmoid functions such as logistic or Gaussian cumulative distribution functions Gompertz (1825); Darroch and Baker (1990); Zwietering et al. (1990). These functions are characterized by a few parameters that are given a biological meaning such as latency period, growth rates, or saturation levels. Disease progress curves are not an exception Madden and Campbell (1990), the repeated assessments performed on a plot can be fitted to a sigmoid function *via* non-linear regression and then collapsed into the parameters that characterize the regressed curve Chang et al. (2018); Omara et al. (2018); Nyanapah et al. (2020).

AUDPC presents some drawbacks: i) several FHB assessments capturing all stages of disease progress development are required to have a reliable measure of FHB resistance, ii) it yields scores with unusual units (% of severity × time), losing the relationship with both time and FHB severity units and making it difficult for interpretation, and iii) scores obtained from unbalanced number of assessments may be biased due to the inability to capture all stages of the disease development. In fact, thesame phenotypic pattern can yield very different AUDPC scores if assessments are performed just too early or too late during the disease development.

Here, we compare the use of AUDPC as an informative and integrating method to evaluate FHB resistance in field trials, with alternative scoring metrics by i) developing efficient methodologies/scoring metrics that maximize the information gained in each assessment, avoiding an exhaustive phenotype burden and allowing fair comparison of scores across trials, ii) comparing the predictive ability of statistical models when prior information about developmental traits such as plant height and anthesis date isadded to the model, and iii) quantify the ability of these new alternatives to capture partial diseases components *via* simulation.

## 2 Material and methods

### 2.1 Plant material and FHB phenotyping

The WheatSustain winter wheat panel is composed of 230 genotypes (cultivars and breeding lines) covering a wide genetic variability across Europe. We used the breeders’ knowledge and the mean of the coefficient of determination to selectthe training set lines for this experiment Laloë (1993); Isidro y Sánchez and Akdemir (2021). The panel represents cultivars developed through breeding programs from Germany (157), Austria (50), Norway (14), Sweden (4), Denmark (3), Poland (1), and Switzerland (1).

Genomic DNA of the 230 varieties was extracted from 1-week-old seedlings, and sent for sequencing using the TraitGenetic 25K single nucleotide polymorphism (SNP) chip. High-quality markers were kept by removing the markers with > 5% heterozygous or missing calls and with a minor allele frequency of< 5%. A total of 22,354 informative markers were retained after filtering. Missing markers were imputed using a multivariate normal (MVN) - expectation maximization (EM) algorithm (Poland and Rife (2012)).

Field trials were carried out for two years (2020 and 2021) and three locations, Tulln (Austria, University of Natural Resources and Life Sciences, BOKU), Vollebekk (Norway, Norwegian University of Life Sciences, NMBU), and Feldkirchen (Germany, SECOBRA Saatzucht GmbH, Secobra). Genotypes were sown in a randomized complete block design with two replications in Tulln and Vollebekk, and non-replicated trials in Feldkirchen. Weather stations collected meteorological conditions for all environments daily from sowing to harvest.

Trials were artificially inoculated with *Fusarium culmorum* or *F. graminearum* during anthesis/inoculation and FHB disease was evaluated as the percentage of infected spikelets per plot, as an integrated measure for incidence and severity Gerlach et al. (1982). The number of assessments per plot was different in each location (**Table 1**). Developmental traits such as anthesis date and plant height were also measured. To include temperature effects on plant growth and disease development, as well as to consider the temperature variability among years and locations, we estimated the accumulated growing degree days (GDDs) expressed in thermal time units as described in McMaster and Wilhelm (1997). AccumulatedGDDs are then computed as follows:


(1) $$\{\begin{array}{l} GDD_{\left(0\right)}^{l}=0\\ GDD_{\left(t\right)}^{l}=GDD_{\left(t-1\right)}^{\;l}+\max\left[0,\left(\frac{T_{max\left(t\right)}^{\;l}+T_{min\left(t\right)}^{\;l}}{2}-T_{base}\right)\right] \end{array}$$

**Table 1:** Partners’ trial design.

	Location	GPS		Sowing	Avg. Anthesis	FHB Assessment	Avg.
Partner	(Country)	coordinates	Year	date	date	protocol	T^a^ (°C)
						6 times with 4 days	
			2020	21/10/19	01/06/20	interval starting 10	11.2
						days after anthesis	
**BOKU**	Tulln	48°19’12’’N					
	(Austria)	16°04’10’’E				6 times with 4 days	
			2021	14/11/20	11/06/21	interval starting 10	10.4
						days after anthesis	
						Single assessment	
			2020	27/10/19	14/06/20	21-47 days	7.2
						after anthesis	
**NMBU**	Vollebekk	59°39’38’’N					
	(Norway)	10°46’55’’E				Single assessment	
			2021	14/11/20	21/06/21	24-31 days	6.8
						after anthesis	
						3 times with 3-4 days	
			2020	07/10/19	31/03/20	interval starting 242	8.1
						days after sowing	
**Secobra**	Feldkirchen	47°54’27’’N					
	(Germany)	11°50’34’’E				5 times with 6-7 days	
			2021	22/09/20	01/05/21	interval starting 222	7.9
						days after sowing	

where $GDD_{\left(t\right)}^{\;l}$ is the value of accumulated growing degree days on the *t*-th day and *l*-th location, $T_{max\left(t\right)}^{\;l}$ and $T_{min\left(t\right)}^{\;l}$ are, respectively, the maximum and minimum temperature recorded on the *t*-th day and *l*-th locationand *T_base_* is the fixed threshold to increment GDDs. *T_base_* was set 5°*C* in this study for all locations. The disease development curves in GDD units for each trial are shown in **Figure 1**.

**Figure 1:**
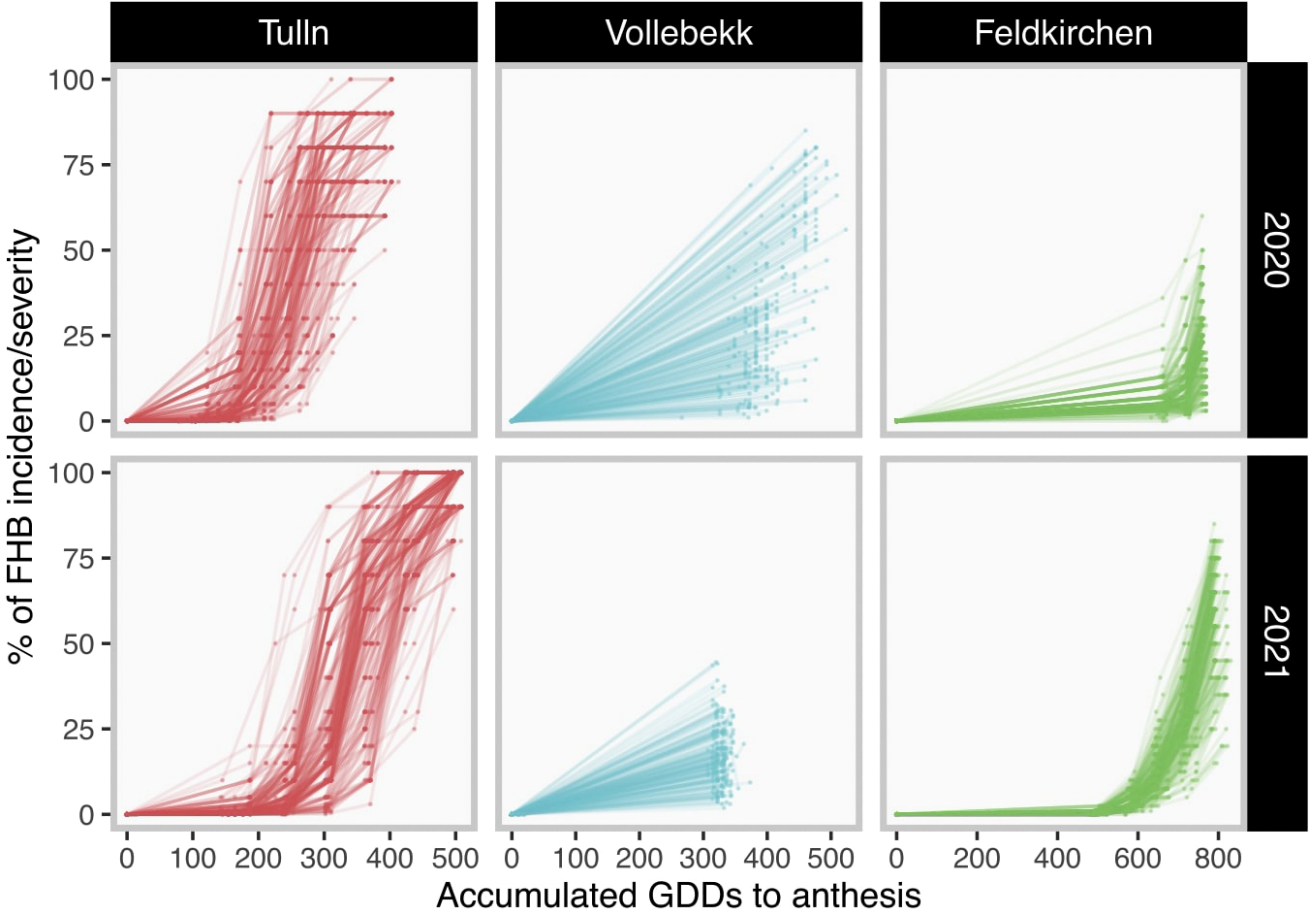
Disease development among trials. Each field trial is graphed in one grid. Grids in the same column have a common location (Tulln, Vollebekk and Feldkirchen, from partners BOKU, NMBU and Secobra, respectively). Grids in the same row have common season (2020 or 2021). Each curve represents the disease development in one plot. Dots represent field assessments and curves are constructed by connecting them. The percentage of FHB incidence/severity is assumed to be null on anthesis.

### 2.2 Scoring metrics

AUDPC is a particular application of Riemans’s sum where a discrete set of *T* assessments are combined to approximate the definite integral between *x*_1_ and *x_T_* of the function that we would ideally obtain if a set of infinite assessments (with a infinitesimal time unit between them) were available. Trapezoidal or mid-point rule Wilcoxson et al. (1975) is the default method to obtain AUDPC but other algorithms may be used to meet the experimental and statistical requirements Simko and Piepho (2012). A summary table of methods used in literature to calculate AUDPC is provided in Jeger and Viljanen-Rollinson (2001).

Here, we propose three alternative scoring metrics with the following formulation. Let X={x_1_, x_2_, …, x_T_}be the set of accumulated Growing Degree Days to Anthesis corresponding to the T assessment’s dates and Y={y_1_, y_2_, …,y_T_} the set of T FHB severity evaluations. We define a scoring metric as a function that maps X and Y into a score to be used as the response variable in further analysis.

#### 2.2.1 Angle

A single-point approach that applies a non-linear transformation, arctan, on the slope computed between a single assessment (*x_T_*, *y_T_*) and the origin (0,0). To integrate more available information, we propose an approach that considers the average time value in which FHB severity reaches the final plateau, instead of just the last assessment. Mathematically:


(2) $$\{\begin{array}{l} \text{Angle}\left(X,Y\right)=arctan\left(\frac{y_{T}}{\frac{x_{p}+x_{T}}{2}}\right)\\ x_{p}=min(X|y=y_{T}) \end{array}$$

where *x_p_* is the minimum value of *X* given that the maximum value of FHB severity (*y_T_*) is reached, i.e. the first time that the maximum disease reached was recorded.

#### 2.2.2 GDD50

We define GDD50 as the accumulated growing degree days in which a plot reaches 50% of the total FHB severity. This concept has been described as the latent period in disease progression in the literature (Das et al. 1993), and has been also used as a parameter to characterize partial disease resistance. We use the following algorithm to compute GDD50 by linear interpolation:


(3) $$\begin{array}{l} \{\begin{array}{l} \text{GDD}50\left(X,Y\right)=\frac{50\;-\;y_{L}}{y_{R}\;-\;y_{L}}\left(x_{R}-x_{L}\right)+x_{L}\\ \{\begin{array}{l} L=arg\;max_{x}(X|y\leq 50)\\ R=arg\;min_{x}(X|y\geq 50) \end{array} \end{array} \end{array}$$

where *L* and *R* are the indexes of the closest points (to the left and right, respectively) to the target disease level (50%), so (*x_L_*, *y_L_*) and (*x_R_*, *y_R_*) are the best points to linearly interpolate GDD50.

#### 2.2.3 Maximum variance (Max *σ* ^2^)

This score is measured as the percentage of FHB severity with the highest variability among plots in the FHB assessment. Mathematically this is expressed as:


(4) $$\{\begin{array}{l} \text{Max }\sigma^{2}\left(X,Y\right)=y_{fhb\_max}\\ fhb\_max=arg\;max_{t}\left[\sum_{i=1}^{N}({\mathrm{FHB}}_{\mathrm{it}}-{\bar{\mathrm{FHB}}}_{.t}{)}^{2}\right] \end{array}$$

where FHB is a *N*×*T* matrix with FHB*_it_* being the disease value for *i*-th plot and *t*-th assessment, and FHB.*_t_* is the mean disease value in the *t*-th assessment. Thus, *fhb*_*max* is the highest variability assessment index. This metric is easy to measure but requires the phenotypic information of all lines before computing it.

### 2.3 Simulation

A stochastic simulation was conducted to obtain a reliable dataset that mimic the same conditions as BOKU’s real database, i.e a set of 6 observations of the disease progress curve for 230 genotypes through 2 years and 2 replicates by year.

Following the phenotypic records from field experiments, we assumed that the disease progress curves follow a sigmoid or logistic behaviour based on two parameters:


(5) $$y=\text{sigmoid}\left(x,a,b\right)=\frac{1}{1+exp\left(\frac{a-x}{b}\right)}$$

where *y* is the FHB incidence/severity recorded for a given *x* value of accumulated GDDs. Parameter *a* determines the position of the curve on x-axis and coincides with both the *inflection point* and the required accumulated GDDs to reach a FHB incidence/severity equal to 50%. Parameter *b* determines the shape of the sigmoid curve and coincides with the reciprocal value of the rate parameter, also known as *intrinsic rate of increase* Madden and Campbell (1990) or *apparent infection rate* Plank (1963). In this simulation we assume no correlation between those parameter and, thus, they are considered to be traits with independent genetic architecture and control.

Real marker information was used to genotype the training population: the simulated genome consisted of 22,354 markers for each trait and marker effects are assumed to randomly follow a normal distribution. True breeding values were calculated by summing effects across all markers and then, scaled to follow a normal distribution (parameter *a*) and a gamma distribution (parameter *b*) as realistic density distributions derived from real data. Populations of *a* and *b* parameters were used to calculate the definite integral values (ideal AUDPC scores).

Components of phenotypic variance (additive genetic variance, environmental variance and residual variance) where chosen to partially disconnect genotypic information and true breeding values and approximate four different heritability values (0.2, 0.5, 0.8 and 1.0). Assessments were performed from days 10 to 30 after anthesis with a period of 4 days between them. Anthesis dates were stochastically computed according to field data.

We also simulated 11 scenarios with different assessment protocols (**Table 2**) where some of the evaluations were considered missing data. We investigated the predictive ability of scoring metrics in all combinations of these two factors (heritability and assessment protocol) resulting in 4×11 = 44 simulated prediction tasks.

**Table 2:** Simulated scenarios.

Scenario	Assessment protocol						No. Assessments
all	1	2	3	4	5	6	6 (100%)
firsts	1	2	3				3 (50%)
mids			3	4	5		3 (50%)
evens		2		4		6	3 (50%)
odds	1		3		5		3 (50%)
limits	1	2				6	3 (50%)
pair 2-5		2			5		2 (33%)
pair 3-6			3			6	2 (33%)
first	1						1 (17%)
fourth				4			1 (17%)
last						6	1 (17%)

BOKU’s design protocol is followed. Assessment are taken from day 10 to 30 after anthesis with a 4-day period. For example, assessments in scenario pair 2-5 are performed 14 and 26 days after anthesis.

### 2.4 Genomic predictions

Predictive ability of scoring metrics was tested by applying Genomic Best Linear Unbiased Predictors (GBLUP) model Van Raden 2008 using the sommer package Covarrubias-Pazaran (2016) in R R Core Team (2021)package [9] in R [44].


(6) {y=Xβ+Zu+ϵ[uϵ]∼N([00],[Kσa2  0  0Rσϵ2])

where *y* is the response variable, i.e. scores. X and Z are known design matrices for fixed (environmental effects, blocks, covariates, etc) and random effects (genotypes or lines). *β* is the vector of regression coefficients of fixed effects (least squares). Vector *u* contains the random genetic additive effects or GEBVs. Random term and residual variances are denoted as $\sigma_{a}^{2}$ and $\sigma_{\epsilon }^{2}$ , respectively. Matrices K and R are the kernels of random effects and residuals to define the covariance structure. K is the additive genomic relationship matrix based on marker information (Endelman and Jannink 2012). Since the model is considered to be homoscedastic (independency between residuals), R = I, the Identity matrix.

To test the performance of predictions based on scoring metrics, we formulated three prediction scenarios using a 5-fold cross-validation scheme. All scenarios involve an independent analysis for each of the four scoring metrics. Scenarios 1 and 2 were applied to field data and only scenario 3 was applied to simulated data.

**Scenario 1.** Whole field data were analyzed simultaneously in a two-step strategy. In the first step, genetic effects (BLUEs) were estimated once by removing environmental effects: 


(7) First step: {y=X1β+X2g+Zu+ϵ[uϵ]∼N([00],[Iσu2  0  0Iσϵ2])

where *y* is the vector with observed phenotype, X_1_ and X_2_ are design matrices for fixed effects, $\hat{\beta }$ is the vector with estimates of trial effects (nested interaction between location and year) and *ĝ* is the vector with estimates for genetic effects (BLUEs). **Z** is the design matrix of random effects and û is the vector with predictions of random effects (nested interaction between trial and replication). Both random terms û and *ϵ* are assumed to be uncorrelated with $\sigma_{\hat{u}}^{2}$ and $\sigma_{\epsilon }^{2}$ as variance components.

In the second step, data was split into train and test according to the cross-validation scheme. BLUEs from training data were used as response variable in four GBLUP models involving different combinations of phenological traits as fixed effects: No covariates (None), Anthesis date (AD), Plant height (PH) and both (AD + PH). The obtained BLUPs (GEBVs). Accuracy values were obtained by correlating predicted GEBVs from unseen lines with BLUEs. This step was replicated 25 times.


(8) Second step:  {g=μ+Xω+Zg*+ϵ[g*ϵ]∼N([00],[Kσg*2  0  0Iσϵ2])

where *ĝ* is the vector with BLUEs from first step, *μ* is the intercept, X is the design matrix for fixed effects, $\hat{\omega }$ is the vector with estimates of covariate effects. Z is the design matrix of random effects and *g* ^*^is the vector with predictions of random effects (GEBVs), which are assumed to be correlated with additive genomic relationship matrix K as covariance structure. $\sigma_{g^{*}}^{2}$ and $\sigma_{\epsilon }^{2}$ are variance components for GEBVs and residuals, respectively.

**Scenario 2.** Field data was split by trial and analyzed independently using a single step strategy and the same combinations of phenological traits as fixed effects. Accuracy values were obtained by correlating predicted GEBVs from unseen lines with phenotypic records. This analysis was replicated 10 times for each of the 6 trial subsets.


(9) Single step:  {y=X1β+X2ω+Zg*+ϵ[ g*ϵ]∼N([00],[Kσg*2  0  0Iσϵ2])

where *y* is the vector with observed phenotype, X_1_ and X_2_ are design matrices for fixed effects, $\hat{\beta }$ is the vector with estimates of replication effects and $\hat{\omega }$ is the vector with estimates for covariate effects. Z is the design matrix of random effects and *g* ^*^ is the vector with predictions of random effects (GEBVs), which are assumed to be correlated with additive genomic relationship matrix K as covariance structure. $\sigma_{g^{*}}^{2}$ and $\sigma_{\epsilon }^{2}$ are variance components for GEBVs and residuals, respectively.

**Scenario 3. **A single step GBLUP model was used to predict GEBVs in each of the 44 simulated predictive tasks:


(10) $$\text{Single step: }\{\begin{array}{l} y=X\beta +Zg^{*}+\epsilon \;\\ \left[\begin{array}{c} \;g^{*}\\ \epsilon \end{array}\right]∼N\left(\left[\begin{array}{c} 0\\ 0 \end{array}\right],\left[\begin{array}{cc} K\sigma_{g^{*}}^{2} & \;0\\ \;0 & I\sigma_{\epsilon }^{2} \end{array}\right]\right) \end{array}$$

where *y* is the vector with observed phenotype, **X** is the design matrix for fixed effects, $\hat{\beta }$ is the vector with estimates of replication and year effects. Z is the design matrix of random effects and *g* ^*^ is the vector with predictions of random effects (GEBVs), which are assumed to be correlated with additive genomic relationship matrix K as covariance structure. $\sigma_{g^{*}}^{2}$ and $\sigma_{\epsilon }^{2}$ are variance components for GEBVs and residuals, respectively.

GEBVs were not only correlated with the observed scored values as shown in previous scenarios: correlations were extended to genetic effects for parameters *a* and *b* and the integral value computed from them. Each predictive task was replicated 30 times. Average accuracy values were calculated and summarized in different ways:

- For each of the 44 predictive tasks, we determined the best performers predicting the four categories (Phenotype, Integral, Parameter *a* and Parameter *b*) and compute the percentage of tasks in which each scoring metric was the best (higher Pearson’s correlation between GEBVs and the respective parameter).
- For each of the 11 scenarios of assessment protocols, we computed the Area Under the Curve (AUC) of the interaction plots resulted by plotting heritability (x-axis) against accuracy (y-axis). The relative performance of alternative scoring metrics (Angle, GDD50, and Max *σ* ^2^) was calculated as the percentage of gain in AUC compared with AUDPC.

## 3 Results

### 3.1 Empirical field data prediction: Scenario 1

**Figure 2:**
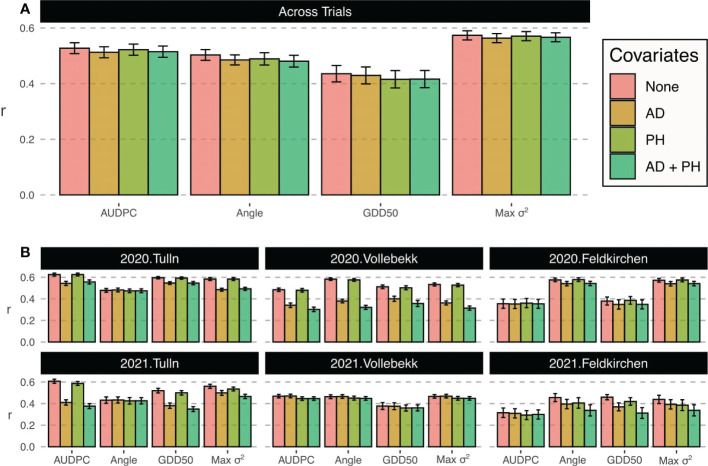
Prediction accuracy (correlation between GEBVs and observed values) for scoring metrics in Prediction scenarios 1 **(A)** and 2 **(B)**. Bar color indicates GBLUP model covariates. Bar height represents average accuracy value and error bars stand for standard deviation.

The prediction accuracy across trials for the scenario 1 is shown in **Figure 2A**. Results indicates that on average AUDPC showed a prediction accuracy of 0.53. Taking as reference this value, the largest accuracies were reached when capturing the maximum variance with a mean accuracy of 0.57, which was +8.74% greater than AUDPC. On the contrary, GDD50 showed the lowest mean accuracy of 0.44 and a decrease of 17.36% compared to AUDPC. The angle metric showed a decrease on average prediction of 4.46% with respect to AUPDC. The use of covariates did not have a significant effect on prediction.

### 3.2 Independent field trials prediction: Scenario 2

**Figure 2B** shows the prediction accuracies within trials. The use of covariates did not have a consistent effect on prediction accuracy. Anthesis or plant height covariates did not improved the average performance, indeed supposed a decrease in prediction in most trials. Results indicate that both locations and years had an important effect on the prediction accuracy and that the number of assessments and disease progress sampling were also important. In general, the maximum variance trait (Max *σ* ^2^) showed greater performance for all years and locations.

In Tulln trials, AUDPC yielded the largest accuracy values, with an average of 0.63 and 0.61 in 2020 and 2021, respectively. GDD50 and Max *σ* ^2^ obtained relatively good accuracy and angle was clearly the worst in both years. In trials where the number of FHB assessments were lower, alternative metrics showed better performance with angle and Max *σ* ^2^ being the most predictable metrics in Feldkirchen 2020 (average accuracy of 0.58) and all alternatives outperforming AUDPC in Feldkirchen 2021 (average accuracy of 0.42). Angle was also the best transformation in Vollebekk 2020 with average accuracy of 0.58 and only GDD50 metric was worse than average in Vollebekk 2021.

### 3.3 Prediction on simulated data: Scenario 3

**Figure 3:**
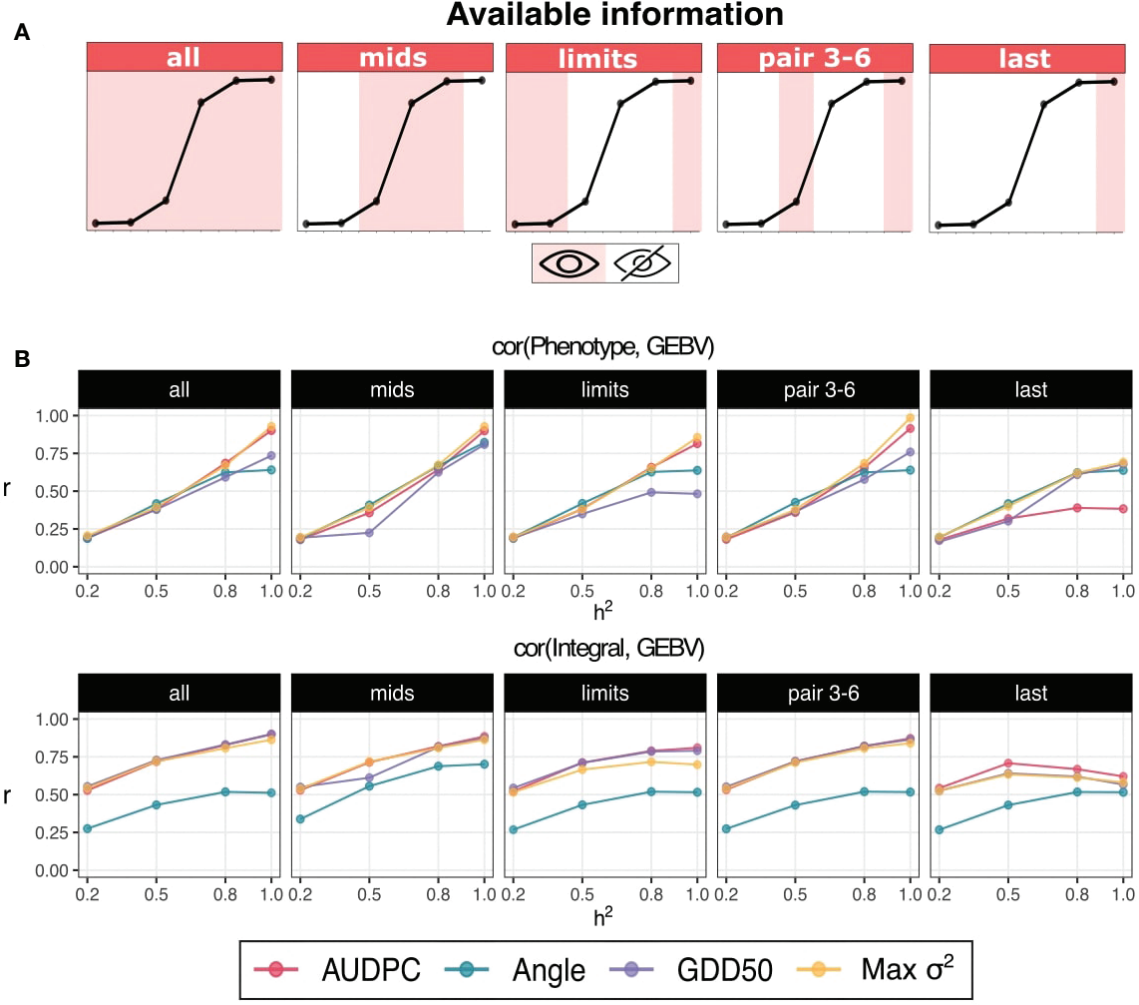
**(A)** Schematic representation of simulated assessment protocols (only 5 from a total of 11 are shown). The curve represents typical disease development with sigmoid behaviour and each point represents a simulated assessment. Shadow and white stripes indicate available and unavailable/missing information, respectively. **(B)** Interaction plots for heritability (x-axis) and accuracy (y-axis). The assessment protocol is denoted in the top of the subplot. Prediction accuracy is computed as the Pearson’s correlation between GEBVs and phenotypes (response variable) in the upper row and integral (ideal AUDPC) in the lower row.

Most representative scenarios for each number of assessment are shown in **Figure 3**: *all* (6), *mids* and *limits* (3), *pair 3-6* (2), and *last* (1). As a general trend, accuracy values increase with higher heritability values. When GEBVs are correlated with phenotype/observed score, best curves and AUC values are obtained by Max *σ* ^2^ and AUDPC: 0.41, 0.40 and 0.41 for scenarios *all*, *mids* and *pair 3-6*, respectively. Highest AUCs are also obtained in these three scenarios when correlating with integral but in that case GDD50 was also a top performer. Although best integral predictions were obtained without restrictions on available data, minimum accuracy losses (<1%) were found when using just the restricted information of assesment protocols *mids* and *pair 3-6*.

Drop accuracy gains for *limits* and *last* were, respectively, -4.8% and -9.1% when predicting phenotype and -5% and -13.3% when predicting integral. Angle has a relative good performance predicting phenotype but has the worst performance predicting integral. AUDPC has a considerable drop in overall accuracy predicting phenotype when only the last point is available (-36.8% of AUC gain respect to accuracy obtained with 6 points).

**Figure 4:**
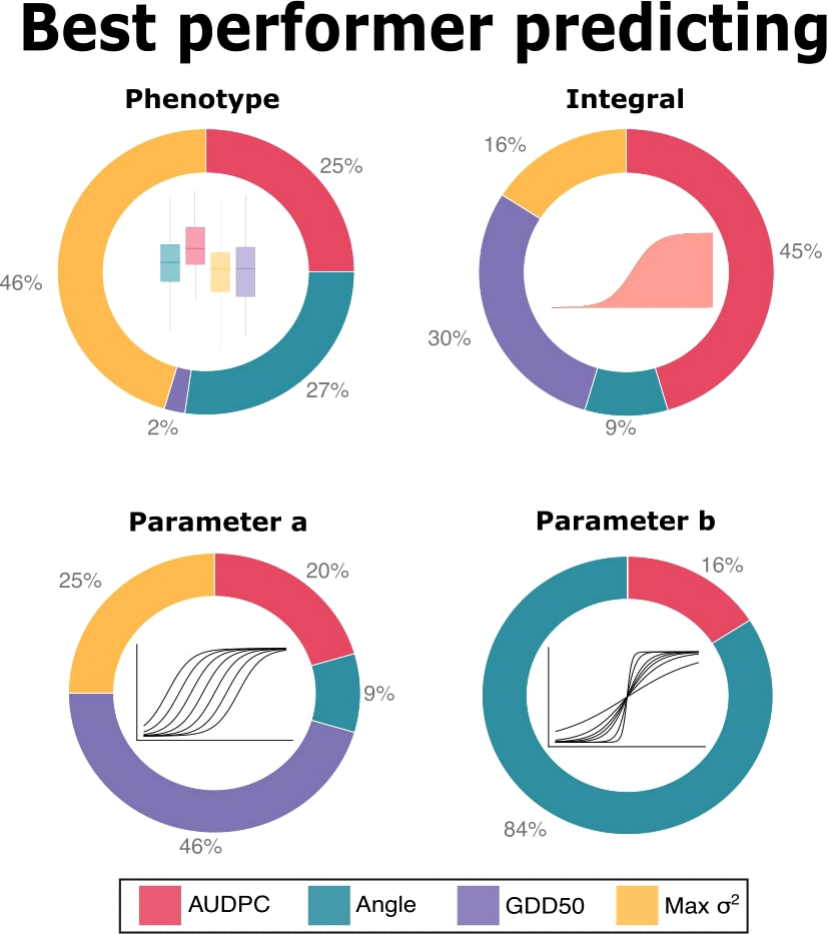
Predictive ability summary. Each chart represents the percentage of tasks in which each scoring metric obtained the best accuracy i.e. the best correlation between GEBVs and the respective parameter (in the top of each subplot).

**Figure 4** shows the percentage of best performers in each task: Max *σ* ^2^ was the best performer predicting phenotype in 46% of predictive tasks. Angle and AUDPC were the best in 27% and 25%, respectively and GDD50 was the best just in 2%. When predicting integral, AUDPC and GDD50 were the best performers, with 45% and 30% respectively. GDD50 was the best performer in almost half (46%) of the predictive tasks for sigmoid parameter *a*. Angle was the best performer in 84% of tasks predicting sigmoid parameter *b* and AUDPC was the best in the remaining 16%.

**Figure 5:**
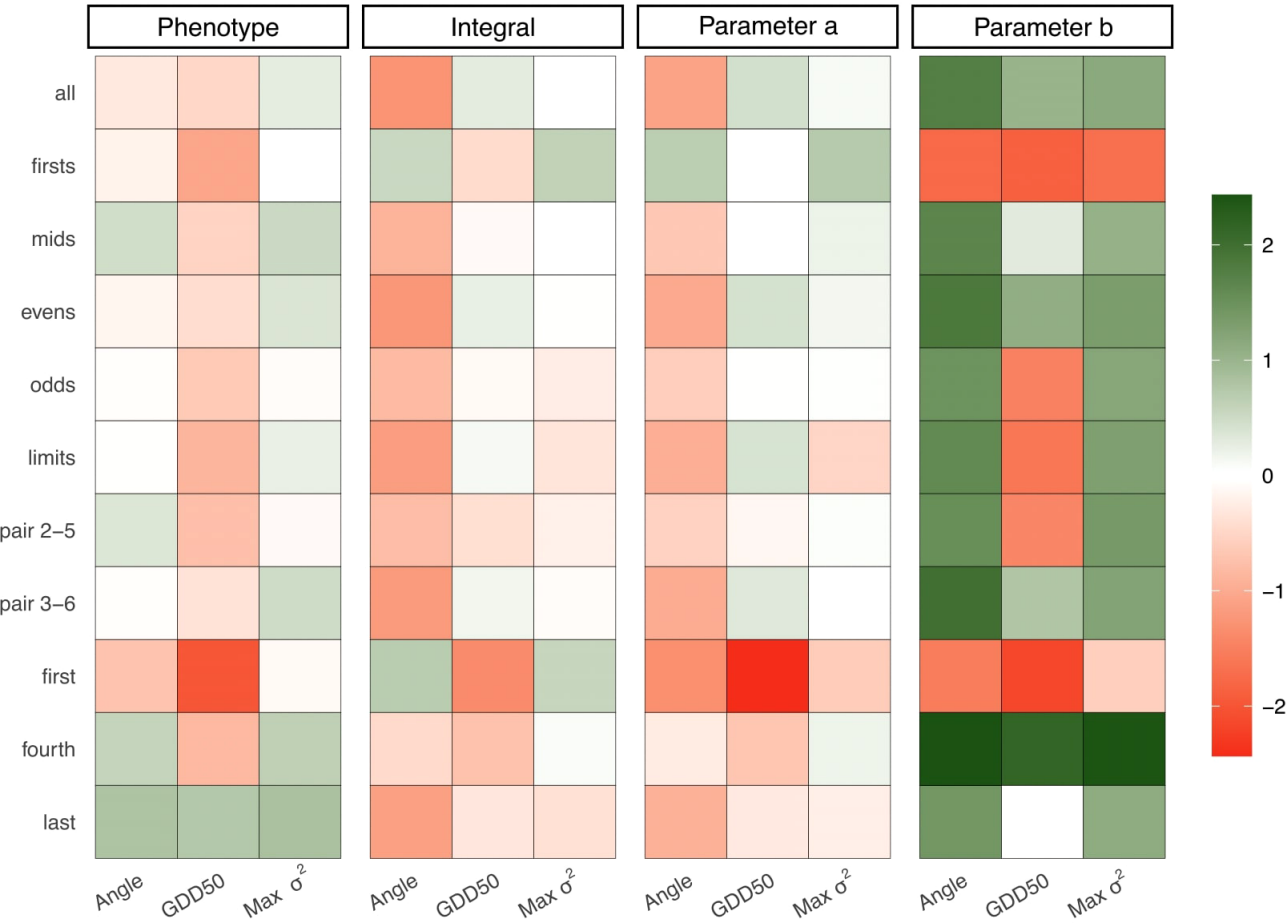
Relative performance of alternative metrics to AUDPC. Each grid represents the percentage of AUC gain of a scoring metric (x-axis) in a determined scenario (y-axis) and parameter (subplot title). Color scale has been normalized to avoid outliers impact on small values (AUC gains range from -86% to +18000%).

**Figure 5** shows the relative performance of alternative methods as AUC gains respect to AUDPC. Max *σ* ^2^ outperformed AUDPC predicting phenotype in 7 out of 11 assessment protocols, including when all the information is available. Results showed similar AUC gain patterns for integral and parameter *a*. Alternative methods, and especially Angle, perform better predicting parameter *b* except in those protocols that capture just the early stages of disease development (*first* and xtit*first*s). GDD50 was better predicting the integral and parameter *a* in four assessment protocols (*all*, *evens*, *limits* and *pair 3-6*) but was penalized in those where only the first stages of disease development werecaptured. All alternative scoring metrics outperform AUDPC predicting phenotype when just the fourth or last assessment is available. Data from **Figure 5** is computed from the absolute values that are shown in the **Supplementary Table S1**.

## 4 Discussion

Besides the common issue of accurately characterizing the disease pattern, the research of FHB resistance has the temporal component factor issue, i.e. since disease patterns changes over time, multiple evaluations of plants/plots are required to properlyquantify disease resistance. This imposes an important drawback in plant breeding programs because i) breeders cannot always measure the disease progression across the crop developmental phases, and ii) increase phenotyping cost and time. Here, we proposed alternative metrics to AUDPC with the aim to i) cope with multi-trial, unbalanced data with different experimental conditions, and ii) yield scores that are fair, comparable, and easy to interpret so they can lead to the elaboration of guidelines for less time-and-labor-consuming but equally informative phenotyping.

### 4.1 Metrics’ predictability

In this work, we assumed that a better understanding of reality translates into higher accuracy when predicting the score value (phenotype) of unseen lines *via* cross-validation. Although AUDPC was expected to reach the best accuracy values in trials with higher number of assessments, the non-optimal results of Feldkirchen 2021 (**Figure 2B**) with 5 assessments suggest tha it is not only the quantity but the quality of those assessments, which should sample all disease stages to compute a representative AUDPC score.

GDD50 was adapted from the well-known parameter “latency period” Darroch and Baker (1990); 314 Ringer et al. (1995); Nyanapah et al. (2020) as a measure of the lag period from infection to the appearance of visible symptoms Das et al. (1993). The characteristics of GDD50 are that i) scores are given in time units and have an easy interpretation, i.e. the time required to reach a half of maximum disease, ii) the highest virulence rate (increase of disease per time unit) is reached when x = GDD50 and iii) GDD50 computation is very sensitive to the assessment protocol and takes advantage of the number and resolution of assessments. In this sense, a strategy based on the evaluation of a few assessments around the set of dates near 50% of infection would allow breeders to reduce the number of assessments without a high penalization in genomic prediction accuracy. For instance, our results demonstrated that GDD50 was strongly penalized in those trials where the disease assessment did not cover both sides around the 50% of FHB infection (Vollebekk in both years and Feldkirchen 2020, **Figures 1**, **2B**). Also, we observed that GDD50 was negatively correlated with all other metrics including AUDPC (**Supplementary Figure S1**). This inversely related correlation can be explained due to the fact that the area under the curve of a sigmoid is asymmetric with respect to the x-axis, which is much greater on the right side or, in other words, after the inflection point (GDD50). Therefore, given a fixed interval to measure AUDPC, the higher the inflection point, the more important part of the area moves outside the measurement window, i.e. the 0-30 days interval used in this work.

The area under the curve of a sigmoid is asymmetric with respect to the x-axis, which is much greater on the right side or, in other words, after the inflection point (GDD50). Therefore, given a fixed interval to measure AUDPC (0-30 DAYS), the higher theinflection point, the more important part of the area moves outside the measurement window (the interval from 0 to 30).

Angle scoring metric was conceived as a straightforward approach which minimum requirements, i.e. just one informative datapoint but with the ability to keep the characteristic non-linearity of the disease progress curve by applying arctan function. Its drawbacks are the inability of integrating more information when it is available and the uninformative nature of its units (degrees or radians) when brought to the field. Here, angle emerged as the most predictable metric when just one point was provided (**Figure 2B**), suggesting that its transformation is the most efficient way to integrate one single datapoint.

With the aim to determine the role of capturing variance on predictability, we calculate the assessment with the maximum variance of the FHB evaluation (Max *σ* ^2^). This metric was computed *a posteriori*, i.e. many different assessments may be performed to calculate Max *σ* ^2^, although just one would be finally used. When field design implies just one assessment, Max *σ* ^2^ is equivalent to just consider the disease percentage and drop the time information. Our results indicated thatMax *σ* ^2^ yielded optimally across and within trials indicating that metrics that capture variance are very informative. Moreover, simulation results indicate that, for higher heritability, there is a positive correlation between predictive accuracyand coefficient of variance (CV), as shown in **Supplementary Figure S2**. We also found that platykurtic phenotypic distributions (thinner tails and less outliers than normal distribution) were linked to better predictions.

To elucidate if the same results patterns was observed in controlled environments, we performed a simulation study which mimicked the disease progression of the BOKU design. Our simulation supported the empirical results (**Figures 3**–**5**), where we found similar prediction performance patterns. Max *σ* ^2^ was the most predictable metric and GDD50 the least predictable one. In addition, alternative metrics were more predictable than AUDPC in assessment protocols that simulate Vollebekk conditions (*fourth* and *last*, **Figure 5**) indicating that a reduction in data points assessment implies a reduction in predictability of AUDPC but not in other metrics.

### 4.2 Developmental traits as covariates

We found that the addition of covariates, such as PH and AD, as fixed effects in the statistical model did not improve prediction accuracy (**Figure 2**). Although it has been demonstrated previously that PH is negatively correlated with FHB traits Michel et al. (2016); Miedaner et al. (2017), other studies have shown that positive, negative, and null correlations could be found in European wheat collections with other traits such as flowering date Buerstmayr et al. (2008). This indicates that the use of covariates does not always translate to an improvement in precision in GS. Nevertheless, the addition of developmental information may be key in other data-driven analyses such as GWAS, where potential spurious associations between molecular markers and FHB traits should be corrected He et al. (2016).

### 4.3 Trade-off between phenotyping effort and accuracy gain

The idea of reducing assessment number to compute resistance scores is not new. Based on the sigmoid behaviour of disease progress curve, and assuming that integral is the target value, Jeger & Viljanen-Rollinson Jeger and Viljanen-Rollinson (2001) proposed that an enough informative AUDPC score for stripe rust severity could be obtained by computing it from the two most informative datapoints (taken at the start of an epidemic and at the end or at a critical growth stage). They obtained Spearman’s correlation values between 0.83 and 0.96 when comparing this approach with the AUDPC scores computed using all available information (7-8 assessments).

We demonstrate that AUDPC values computed from two or three targeted datapoints (critical growth stage and end of epidemic) are enough informative to obtain both a predictable phenotype and a good approach of the integral (**Figure 3**). This approach would reduce evaluation or phenotyping effort with minimal accuracy losses. Also, we found that GDD50 is a strong candidate to compute integral when those targeted datapoints (second, third and sixth assessments) are provided, as shown in **Figures 3B**, **5** and **Supplementary Table S1**. These points are crucial in the GDD50 computation because they increase the probability of finding values in both below and above 50% of disease, as shown in **Supplementary Figure S3**.

### 4.4 Capturing sigmoid parameters

Patterns of disease development over time are usually well-fitted to nonlinear models Ratkowsky (1993). Nonlinear functions are characterized by few parameters which determine key features of each individual curve. We can assign a biological meaning to these parameters and use them as scoring values for partial disease components as interesting sources of quantitative disease resistance. For example Nyanapah et al. Nyanapah et al. (2020) compared eight measures of disease resistance to gray leaf spot on maize which include latency period, rate parameter, and AUDPC variants such as standard AUDPC and AUDPC to inflection point. Chang et al. Chang et al. (2018) compared the goodness of fit of four different nonlinear models (exponential, monomolecular, logistic and Gompertz) to cocoa black pod progress curve. Experimental procedures in these studies implied the use of a manageable number of genotypes and exhaustive phenotyping benchmark that allow nonlinear least squares optimization methods to properly fit datapoints to curves.

In our approach, we applied this knowledge to the problem of characterizing disease curves where datapoints were not enough to use standard nonlinear fitting procedures. Simulation experiment allowed us to understand the role of each scoring metric on thecapture and prediction of the partial disease components reflected in the sigmoid function such as the latency period (inflection point or parameter *a*) which can be targeted by computing GDD50 and the apparent disease rate (rate parameter or parameter *b*), which was successfully captured by our Angle approach. Due to this findings, we suggest further analysis to properly understand the basis of these relations and to transfer this knowledge to field trials and real selection processes.

## 5 Conclusions

In this study, we tested the ability of scoring metrics (AUDPC, Angle, GDD50, and Max *σ* ^2^) to efficiently capture and integrate field information. Our findings demonstrated that if the field assessments capture critical growth stages of disease development such as the inflection point and/or the end of the disease growth, then a reduction in the number of assessments did not imply a significant predictive accuracy loss. Field assessments protocols that try to capture maximum variance are a great approach to characterize quantitative resistance. GDD50 is a feasible alternative to measure FHB resistance as soon as disease sampling covers 50% of the disease infection. Thus, historical data analysis could be performed to predict the most optimal time to measure FHB in the field experiment, due tothe predictive role of GDD measures. In addition, alternative metrics such as GDD50 and Angle are good approaches to compute sigmoid parameters, which can be translated into quantitative resistance components without the usual requirements of data availability in non-linear fitting procedures. We propose the following guideline for phenotyping FHB resistance:

◼ If requirements of resolution and sampling are met (i.e. phenotyping assessment is not an issue), characterizing FHB resistance as the percentage of infected spikelets per plot when variance is high is a great approach for the prediction of unseen genotypes.
◼ If the number of assessments is restricted but a targeted strategy is possible, capturing inflection point is essential to characterize disease progress curve. Following our field data, the optimal measurements should be carried out between 20 and 30 days after anthesis/inoculation.

## Data availability statement

The original contributions presented in the study are included in the article/**Supplementary Material**. Further inquiries can be directed to the corresponding authors.

## Author contributions

G-AJ performed statistical analyses, wrote large part of the article and prepared the figures. I-SJ conceived the study, and wrote a large part of the article. LM, BH, I-SJ, and HL conceived the project and obtained fundings. I-SJ and G-AJ drafted the manuscript. LM, ML, MS, HL and BH, collected and performed initial quality check of the field data. All authors discussed the results and reviewed the manuscript. All authors contributed to the article and approved the submitted version.

## Funding

The WheatSustain project was carried out under the ERA-NET Cofund SusCrop (Horizon 2020 Grant No 771134), being part of the Joint Programming Initiative on Agriculture, Food Security and Climate Change (FACCE-JPI). I-SJ was supported by the Beatriz GalindoProgram (BEAGAL18/00115) from the Ministerio de Educación y Formación Profesional of Spain and the Severo Ochoa Program for Centres of Excellence in R&D from the Agencia Estatal de Investigación of Spain, grant SEV-2016-0672 (2017-2021) to the CBGP. G-AJ is working under a UPM predoctoral grant as part of the programme “Programa Propio I+D+i” financed by the Universidad Politécnica de Madrid. ML, MS, and BH were supported by the Austrian Federal Ministry of Agriculture, Regions and Tourism (grantnumber DaFNE-101402). LM was supported by the Research Council of Norway (NFR grant 299615), and and HL by Deutsches Bundesministerium für Bildung und Forschung (031B0810).

## Conflict of interest

Author HJ was employed by Secobra Saatzucht GmbH.

The remaining authors declare that the research was conducted in the absence of any commercial or financial relationships that could be construed as a potential conflict of interest.

## Publisher’s note

All claims expressed in this article are solely those of the authors and do not necessarily represent those of their affiliated organizations, or those of the publisher, the editors and the reviewers. Any product that may be evaluated in this article, or claim that may be made by its manufacturer, is not guaranteed or endorsed by the publisher.

## Supplementary material

Supplementary Figure 1 Correlation between scoring metrics in field trials. Diagonal grids show the normalized distribution of each score split by trial. Lower triangle grids show the pairwise scatter plots between the four scoring metrics. Upper triangle grids indicate the pairwise overall correlation (in gray) and the trial correlations between the four scoring metrics. Trials from Tulln, Feldkirchen and Vollebekk are denoted, respectively, by colors red, green and blue. Year 2020 is denoted with darker colors.

Supplementary Figure 2 Correlation between the coefficient of variation (CV) of phenotypic distributions and the predictive accuracy (r) when all simulated information is available. Color and shape determine, respectively, the scoring metric and the heritability. Each dot represent one replicate (out of 30).

Supplementary Figure 3 Disease stages coverage in each simulated assessment protocol. Each grid represents the disease development curves of simulated plots given the available information of the assessment denoted above. Dots represent simulated assessments and curves are constructed by connecting them. Red color indicate that there is no available information below 50% of disease in that plot and blue color indicate that there is no available information above 50% in that plot. Gray curves contains at leastone value below and above 50%.

Supplementary Table 1 Absolute AUC values obtained from the heritability-accuracy interaction such those shown on . Row and column names denote, respectively, the assessment protocol and the scoring metric. Subtitles denote the value which is being predicted. Underlined AUC scores are the higher in each assessment protocol and predicted parameter.
