# Supplementary figures and images for: Alternative scoring methods of fusarium head blight resistance for genomic assisted breeding
Source: Front Plant Sci. 2023 Jan 11;13:1057914. doi: 10.3389/fpls.2022.1057914 (PMC9876611; doi:10.3389/fpls.2022.1057914)

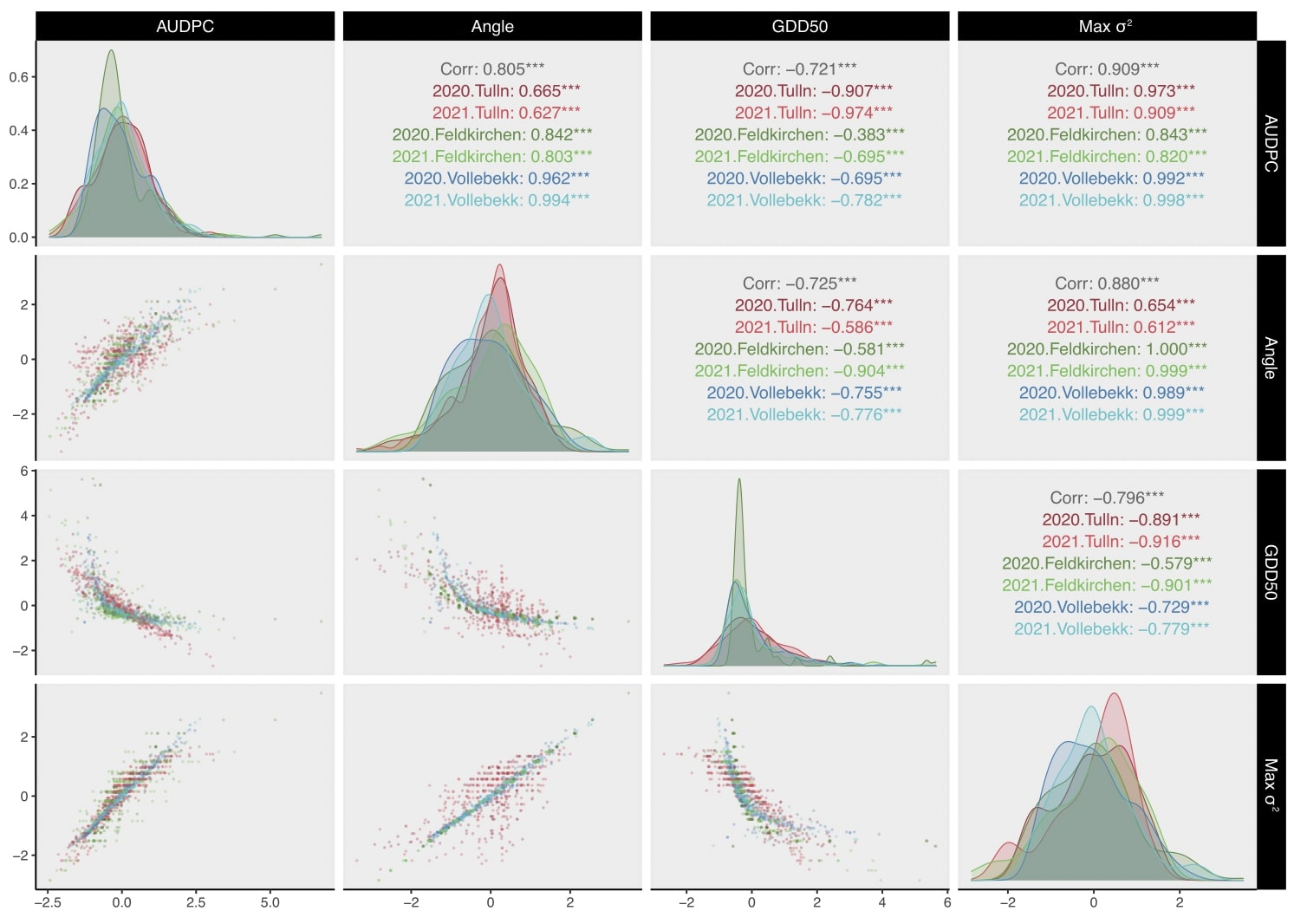

Supplement: Supplementary Figure 1 — Correlation between scoring metrics in field trials. Diagonal grids show the normalized distribution of each score split by trial. Lower triangle grids show the pairwise scatter plots between the four scoring metrics. Upper triangle grids indicate the pairwise overall correlation (in gray) and the trial correlations between the four scoring metrics. Trials from Tulln, Feldkirchen and Vollebekk are denoted, respectively, by colors red, green and blue. Year 2020 is denoted with darker colors. [file Image_1.jpeg]

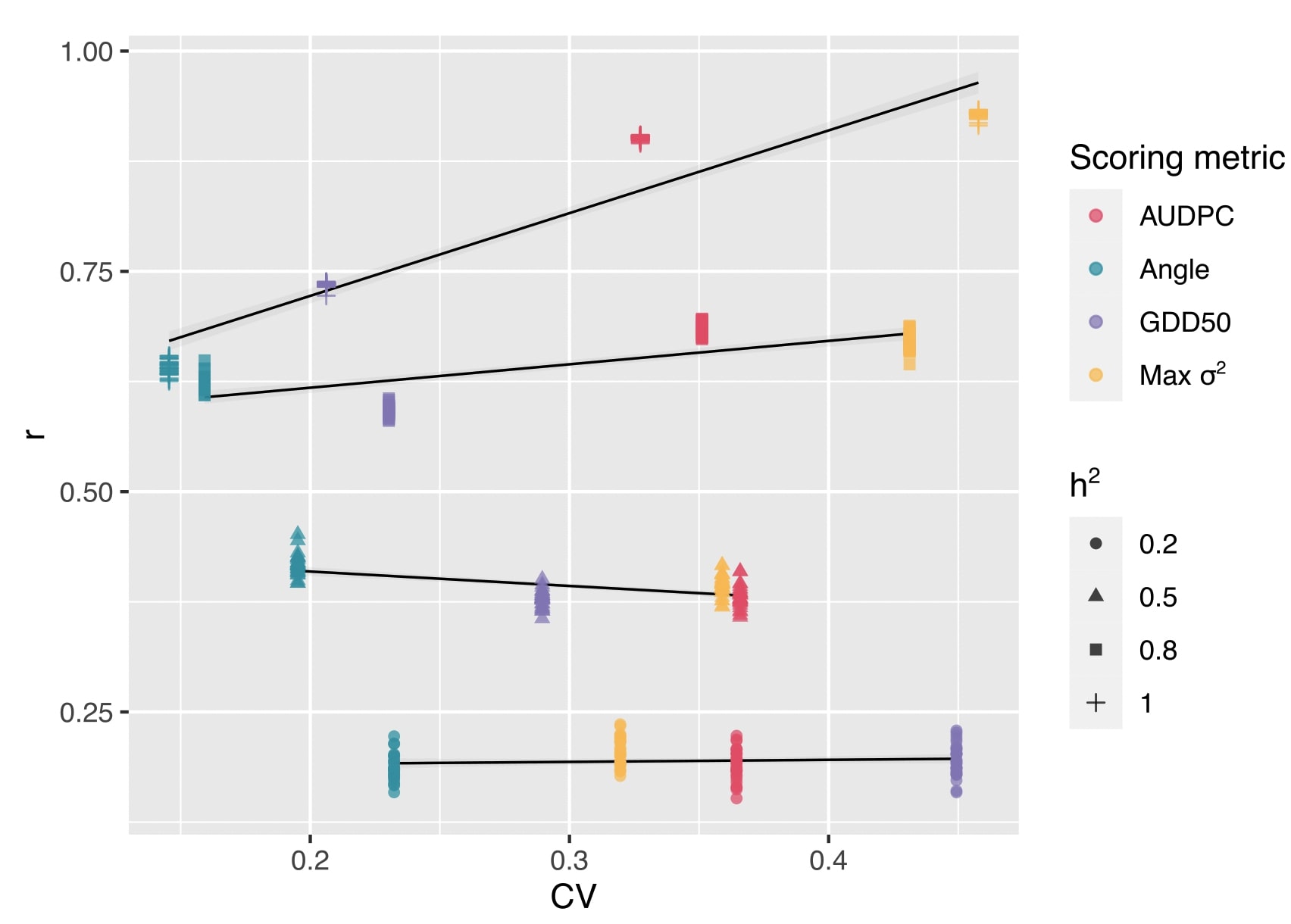

Supplement: Supplementary Figure 2 — Correlation between the coefficient of variation (CV) of phenotypic distributions and the predictive accuracy (r) when all simulated information is available. Color and shape determine, respectively, the scoring metric and the heritability. Each dot represent one replicate (out of 30). [file Image_2.jpeg]

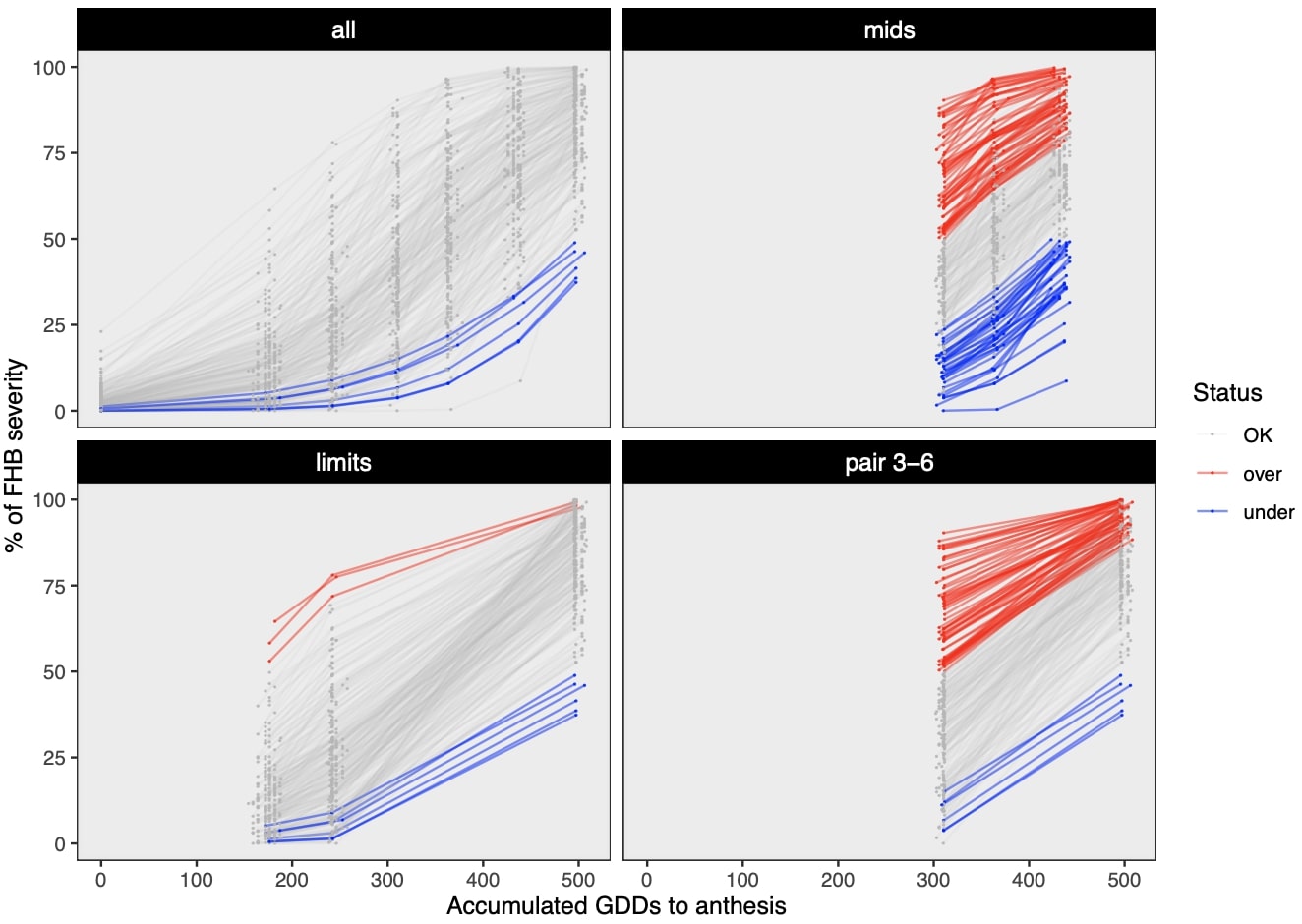

Supplement: Supplementary Figure 3 — Disease stages coverage in each simulated assessment protocol. Each grid represents the disease development curves of simulated plots given the available information of the assessment denoted above. Dots represent simulated assessments and curves are constructed by connecting them. Red color indicate that there is no available information below 50% of disease in that plot and blue color indicate that there is no available information above 50% in that plot. Gray curves contains at leastone value below and above 50%. [file Image_3.jpeg]

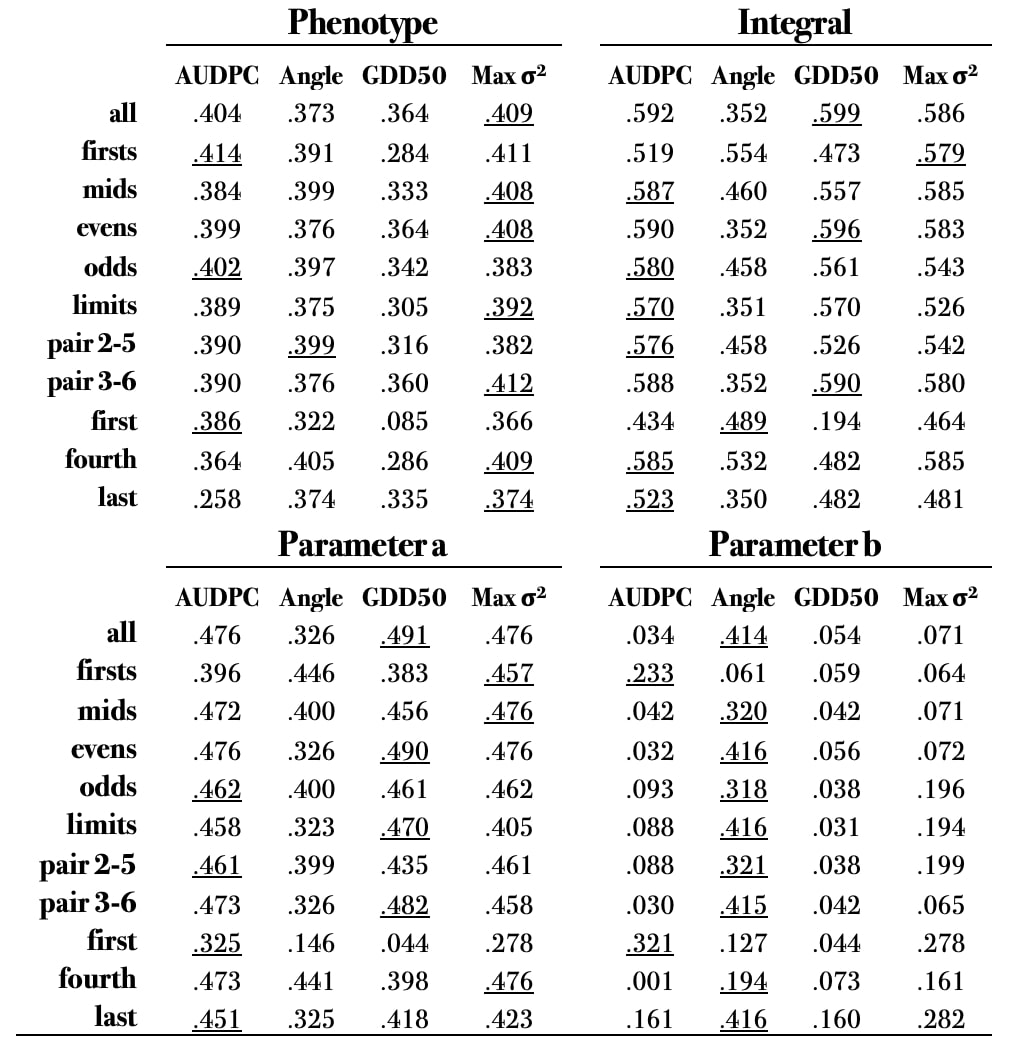

Supplement: Supplementary Table 1 — Absolute AUC values obtained from the heritability-accuracy interaction such those shown on . Row and column names denote, respectively, the assessment protocol and the scoring metric. Subtitles denote the value which is being predicted. Underlined AUC scores are the higher in each assessment protocol and predicted parameter. [file Image_4.jpeg]
